# Neuroscience Informed Prolonged Exposure Practice: Increasing Efficiency and Efficacy Through Mechanisms

**DOI:** 10.3389/fnbeh.2018.00281

**Published:** 2018-11-20

**Authors:** Monika M. Stojek, Lauren B. McSweeney, Sheila A. M. Rauch

**Affiliations:** ^1^Department of Psychiatry, Emory University School of Medicine, Atlanta, GA, United States; ^2^Emory Healthcare Veterans Program, Atlanta, GA, United States; ^3^Atlanta VA Medical Center, Atlanta, GA, United States

**Keywords:** PTSD, prolonged exposure, psychotherapy, exposure therapy, neuroscience, extinction learning

## Abstract

Prolonged exposure (PE) is an empirically supported efficacious treatment for posttraumatic stress disorder (PTSD). In this focused review, we briefly review the neurobiological networks in PTSD relevant to PE, discuss the theoretical basis of PE, review the neurobiological mechanisms underlying the effectiveness of PE and identify the enhancements that can be applied to increase treatment response and retention. Based on the reviewed studies, it is clear that PTSD results in disrupted network of interconnected regions, and PE has been shown to increase the connectivity within and between these regions. Successful extinction recall in PE is related to increased functional coherence between the ventromedial prefrontal cortex (vmPFC), amygdala and the hippocampus. Increased connectivity within the dorsolateral PFC (dlPFC) following PE is associated with more effective downregulation of emotional responses in stressful situations. Pre-existing neural connectivity also in some cases predicts response to exposure treatment. We consider various enhancements that have been used with PE, including serotonin reuptake inhibitors (SSRIs), D-cycloserine (DCS), allopregnanolone (ALLO) and propranolol, repetitive transcranial magnetic stimulation (rTMS), oxytocin and MDMA. Given that neural connectivity appears to be crucial in mechanisms of action of PE, rTMS is a logical target for further research as an enhancement of PE. Additionally, exploring the effectiveness and mechanisms of action of oxytocin and MDMA in conjunction with PE may lead to improvement in treatment engagement and retention.

Posttraumatic stress disorder (PTSD) has been increasingly recognized as a public health concern, with increased visibility as military service members return from combat deployments. Due to the psychological burden associated with its symptoms, PTSD is associated with significant physical, psychosocial and economic hardships (Ramchand et al., 2015). The hallmark symptoms of PTSD include: (1) re-experiencing the memory of the traumatic event through thoughts, images or nightmares; (2) avoidance of the reminders of trauma; (3) negative self-image and/or deterioration in mood; and (4) physiological hyperarousal (American Psychiatric Association, 2013). Trauma focused therapies, such as exposure-based and cognitive therapies, particularly prolonged exposure (PE) and cognitive processing therapy (CPT), are efficacious for the treatment of PTSD (Watts et al., 2013) and are recommended in professional treatment guidelines (American Psychological Association, 2017; Department of Veterans Affairs & Department of Defense (VA & DoD), 2017) and the Institute of Medicine Report (Institute of Medicine, 2014). PE has been studied extensively and has consistently demonstrated causal reduction of symptoms of PTSD, anxiety and depression (Watts et al., 2013). In the era of precision medicine (Shukla et al., 2015) and objective (e.g., neurobiological and psychophysiological) measures of treatment outcome, it is crucial to move beyond establishing efficacy of treatment approaches and to investigation of the mechanisms of action of such efficacious treatments. A focus on mechanisms of action will contribute to increasing efficacy (remission and response), efficiency (time to response) and access (moving the active ingredients to new models of application).

In this review article, we will bridge two bodies of knowledge: the clinical mechanisms of action of an empirically-supported treatment for PTSD and the neurobiological underpinnings of such intervention. As such, we are bridging the bench and the bedside by writing for clinicians who provide these interventions and for the researchers designing future studies. We will begin by briefly reviewing neural circuits implicated in PTSD that are relevant in PE, followed by a description of PE and its theoretical underpinnings. We will examine the state of current knowledge on how PE impacts the neural circuits related to PTSD. We will provide a synthesis of findings to date and discuss neurobiological enhancements that have been or may be used in conjunction with PE to enhance its effectiveness. Finally, we will offer some neurobiologically-informed directions for future research and practice.

## Neurobiology of PTSD

In recent years, several reviews have summarized the state of knowledge regarding neural circuits implicated in PTSD (Duval et al., 2015; Liberzon and Abelson, 2016; Sheynin and Liberzon, 2017). In the current article, we briefly review the circuits most relevant to understanding therapeutic mechanisms of action in PE.

The neural circuit underlying fear-related learning is most commonly implicated in models of PTSD (Rauch et al., 2006; Shin and Handwerger, 2009; Jovanovic and Ressler, 2010; Shvil et al., 2013). The fear neurocircuitry consists of several brain structures including the amygdala, anterior cingulate cortex (ACC) and the ventromedial prefrontal cortex (vmPFC; Shin and Liberzon, 2010). The fear network is implicated in evaluating whether a stimulus should be approached or avoided, and activity in this network is correlated with anxiety (Shin and Liberzon, 2010). The evidence from fMRI studies indicates that the amygdala, which receives sensory input and orchestrates the response to threatening signals, is overactive in PTSD, likely contributing to the exaggerated fear response and re-experiencing symptoms (Rauch et al., 2006; Shin et al., 2006; Milad et al., 2009). The vmPFC downregulates the amygdala and appears to play a critical role in extinction recall (Quirk et al., 2000). In PTSD, vmPFC is hypoactive, thus projecting less inhibitory input and contributing to the hyperactivation of the amygdala (Rauch et al., 2006; Shin et al., 2006; Liberzon and Abelson, 2016). ACC which, along with the amygdala, processes aversive stimuli and projects to the peripheral nervous system to trigger a response, has been shown to be hyperactive during extinction recall in individuals with PTSD (Hayes et al., 2012; Koch et al., 2016). This dysregulation in the fear neurocircuitry is purported to underlie the failure to extinguish the fear response over time (Rauch et al., 2006; Jovanovic and Ressler, 2010; Liberzon and Abelson, 2016) and possibly the overgeneralization of fear to non-threatening cues (Stevens et al., 2013; Lopresto et al., 2016).

The neurocircuitry implicated in context processing has also received attention in relation to PTSD etiology and maintenance (Liberzon and Abelson, 2016). Within the fear neurocircuitry, hippocampus is involved in the process of contextualization, or accurately discriminating threat in the environment (Maren and Fanselow, 1995). Hippocampal inputs provide contextual information to the amygdala and to the vmPFC, thus downregulating the amygdala and facilitating extinction learning as the network normally functions (LeDoux, 2000). In the overgeneralized conditioned fear response present in PTSD, hippocampus and vmPFC are hypoactive in environments that are safe thus projecting dampened inputs to the amygdala and failing to downregulate amygdala’s functioning in those contexts (Garfinkel et al., 2014). Hypoactivity of the vmPFC and the hippocampus may contribute to the re-experiencing symptoms via difficulties in extinction learning, a process further reinforced by avoidance (Pitman et al., 2012). Individuals with PTSD have demonstrated difficulty in maintaining learned fear extinction, or extinction recall (Milad et al., 2008, 2009). In fMRI studies, patients with PTSD have reduced hippocampal and vmPFC activation, and increased ACC activation during extinction recall (Milad et al., 2009; Hayes et al., 2012; Shvil et al., 2014; Koch et al., 2016) and the contextual processing period of extinction recall (Rougemont-Bücking et al., 2011). Previous studies have found that smaller hippocampal volume is associated with PTSD (Gilbertson et al., 2002). Hippocampus volume does not appear to change longitudinally over the course of PTSD in adults (Lindgren et al., 2016) but there is some evidence for impaired hippocampus development during childhood maltreatment (Dannlowski et al., 2012; Keding and Herringa, 2015). This suggests that hippocampal volume is vulnerability factor for PTSD that is likely epigenetically shaped.

Finally, emotion regulation deficits, or difficulties in awareness and modulation of intense negative emotional states, may be a transdiagnostic factor for the development and maintenance of many psychological disorders, including PTSD (Bradley et al., 2011; Sheynin and Liberzon, 2017). Based on neuroimaging findings, two broad types of emotion regulation include explicit and implicit emotion regulation (Gyurak et al., 2011). Explicit emotion regulation is effortful and requires some level of insight and awareness (Etkin et al., 2015). The most known example is reappraisal, or an alteration of the meaning of an emotion-inducing stimulus. The brain regions implicated in reappraisal include dorsolateral PFC (dlPFC), ventrolateral PFC (vlPFC) and parietal cortex (Buhle et al., 2014; Kohn et al., 2014). Implicit emotion regulation is automatic in response to a stimulus and can occur without insight or awareness (Gyurak et al., 2011). The vmPFC is the brain region primarily implicated in implicit emotion regulation (Gyurak et al., 2011). Therefore, a neurobiological emotion regulation model posits that the PFC regions are responsible for cognitive control via stimulus interpretation (either explicit or implicit), thus downregulating the amygdala activation in response to emotionally salient stimuli (Ochsner et al., 2012; Buhle et al., 2014). Only a handful of studies examined neuropsychological and neurobiological correlates of emotion regulation in PTSD (New et al., 2009; Aupperle et al., 2012; Rabinak et al., 2014; Shepherd and Wild, 2014). The results point to the pattern of suppressing emotions, using less cognitive reappraisal (Shepherd and Wild, 2014), deficits in inhibitory control (Aupperle et al., 2012), and diminished downregulation of emotional response including hypoactivation of dlPFC (New et al., 2009; Rabinak et al., 2014). While these three broad neural circuits are not an exhaustive representation of neurobiological underpinnings of PTSD, they comprehensively represent the regions of interest when considering the effects of PE on neural activity.

## Prolonged Exposure and its Theoretical Underpinnings

PE (Foa et al., 1991) is an exposure-based psychological intervention designed to treat PTSD following trauma. The main goal of PE is to promote emotional processing through deliberate systematic confrontation with trauma-related stimuli (Foa, 2011). The key components of PE are: (1) repeated imaginal exposure (IE), which requires the individual to revisit their trauma memory in a therapeutic context; (2) *in vivo* exposure (IVE) to places and situations that are avoided because they evoke stress and anxiety; and (3) emotional processing that focuses on reviewing the experience of exposure and its impact on thoughts related to the trauma. A significant body of evidence has demonstrated the efficacy and effectiveness of PE in the treatment of PTSD and related depression, general anxiety, guilt, anger, and physical health concerns (e.g., Foa and Rauch, 2004; Rauch et al., 2009). In relation to PTSD, PE has reliably established clinically significant reduction in symptoms, with large effect sizes among various treatment samples with a variety of trauma histories (Powers et al., 2010). Based on intent to treat analyses, on average, 53% of those who initiate PE no longer meet diagnostic criteria for the disorder, and the rate of diagnostic change increases to 68% among individuals who complete treatment (Bradley et al., 2005).

Development of PE was based on Emotional Processing Theory (Foa and Kozak, 1986). The fundamental tenet of EPT is that there are fear structures (expanded later to include other emotions as well as fear; Rauch and Foa, 2006; expanded later to include other emotions as well as fear; Rauch and Foa, 2006) that include stimulus, response, and meaning elements, and that these structures are there to assist in response to situations of danger or threat. Activation of the adaptive fear structure is viewed as a normal and rational response to a stimulus (e.g., a car racing towards me), meaning (dangerous) that elicits a fear response (increased heart rate), followed by an action (moving out of the way) to remain safe (Foa and Kozak, 1986). Following trauma, additional unhelpful fear (or other emotion) structures develop that represent the stimulus, response, and meaning elements from the time of the trauma but that may not represent actual threat or danger outside of the specific trauma context. Rauch and Foa (2006) state that an optimal level of activation including all elements of the trauma structure is necessary for successful treatment. When the full memory, including all the emotional and cognitive responses, is activated, updated information that is incompatible with the trauma memory can be incorporated (reconsolidated) into the memory structure. Extinction^1^ occurs in the context of repeated exposure to the feared stimulus and is marked by a reduction in physiological and emotional intensity of response to that stimulus (Sripada and Rauch, 2015). They argue that the trauma structure of individuals with PTSD contains maladaptive cognitions that underlie the maintenance of PTSD (Foa et al., 1991).

Theoretically, the learning principles of classical conditioning explain the acquisition of the fear response (Rothbaum and Davis, 2003; Blechert et al., 2007). Specifically, the traumatic event represents the unconditional stimulus (UCS), which produces the unconditioned response (UCR; e.g., fear, helplessness or horror). Neutral stimuli that were present during traumatic event become conditioned stimuli (CS) that can elicit a conditioned response (CR) similar to the reaction during the initial traumatic event. In an effort to reduce the experience of fear (or other negative emotions), individuals will avoid stimuli which evoke the emotional response. Consistent with the operant conditioning model (Mowrer, 1960), this avoidance serves as a negative reinforcement strategy, reducing the experience of negative emotions associated with the CS. In patients with PTSD, the overgeneralization of conditioned fear maintains and exacerbates PTSD symptoms (Lissek and Grillon, 2012).

While EPT and the learning theory are two separate theories of PTSD, there is considerable overlap in the concepts from both theories, and the mechanisms of PE have been conceptualized using both theories (Rauch and Liberzon, 2016). The EPT concept of extinction in PE can be better described in terms of extinction and relearning including contextualization of learning and memory (Rauch and Liberzon, 2016). Extinction occurs when new inhibitory associations are formed on top of the fear associations, and is marked by a reduction in subjective fear response to the feared memory and its reminders. This relearning is facilitated through the process of contextualization, or learning to discriminate between safety and threat cues, depending in the context in which they occur (Maren et al., 2013). The cognitive and emotional processing changes that occur in tandem with extinction and relearning are marked by increased sense of competence, reduced sense that the world as dangerous, and reduction in social and emotional withdrawal (Rothbaum et al., 2005; Rauch and Liberzon, 2016). Although change in self-efficacy and trauma-related beliefs is not central to learning models, this learning of increased competence to cope with negative affect and reduced sense of a dangerous world can be viewed as a form of inhibitory learning (Rauch and Liberzon, 2016), which has been speculated to be a one of the key mechanisms of action in exposure-based treatment for PTSD (Craske et al., 2008).

## Neurobiology of PE

Development of PE was not rooted in a neurocircuitry-based framework but its purported mechanisms of action and effectiveness may be examined using that framework. The fear and contextualization neurocircuitry is implicated in extinction learning and recall, one of the putative active components of PE (Jovanovic and Ressler, 2010; Liberzon and Abelson, 2016; Lopresto et al., 2016). A few early studies which used exposure-based therapy (but not PE specifically) have found increased activation in the prefrontal regions in individuals with PTSD following a course of psychotherapy (Felmingham et al., 2007; Peres et al., 2007). Following therapy, increased activation in the left PFC was correlated with decreased activation of the amygdala and increased activation in the hippocampus during retrieval of the traumatic memory by individuals with PTSD (Peres et al., 2007). During processing of threatening stimuli, individuals with PTSD demonstrated increased vmPFC (particularly rostral ACC) activation and decreased amygdala activation from pre- to post-treatment (Felmingham et al., 2007). Recent studies examined the effect of PE on extinction in laboratory settings. During fear extinction recall paradigm, individuals who underwent PE demonstrated a decrease in rostral ACC activation from pre- to post-treatment (Helpman et al., 2016a). Structurally, those who remitted from PTSD following a course of PE demonstrated volume reduction and thinning in the left rostral ACC, compared to those who did not remit and to controls (Helpman et al., 2016b). No between-group differences in ACC volume and thickness were observed prior to treatment (Helpman et al., 2016b).

One putative explanation of these functional and structural brain changes following PE is the extinction of maladaptive cognitive-emotional connections resulting from extinction learning (Helpman et al., 2016b). In PTSD, fear neurocircuitry may reinforce existing connections and contribute to the formation of new ones (Johansen et al., 2011). These results suggest that effective extinction that occurs during PE contributes not only to a more balanced feedback loop between the vmPFC and the amygdala (Felmingham et al., 2007; Peres et al., 2007) but also to the thinning of the ACC via decreased activation and pruning of the connectivity (Helpman et al., 2016b). This reciprocal relationship between neural connectivity and treatment response may also work the other way. There is some evidence that individual’s capacity to benefit from PE may be modulated by the degree of spontaneous PFC downregulation of the amygdala when processing threat cues prior to treatment (Fonzo et al., 2017). Specifically, patients who before receiving a course of PE had greater activation of the dlPFC as well as less amygdala activation during an emotional reactivity task (detection and processing of threatening cues), showed the biggest gains from PE (Fonzo et al., 2017). This finding potentially suggests that extinction learning and recall may be more difficult for some individuals to achieve based on the extent to which their vmPFC is able to downregulate the amygdala during exposure. Hippocampus is another structure integral to the fear neurocircuitry. Structural differences in the contextualization neurocircuitry, particularly the hippocampus, have been shown to be associated with vulnerability to PTSD (Gilbertson et al., 2002; Lindgren et al., 2016). PE responders and controls had greater baseline hippocampal volume compared to treatment non-responders (Rubin et al., 2016), indicating that hippocampal volume may not only confer risk for PTSD development (Gilbertson et al., 2002; Lindgren et al., 2016) but also be related to better outcome in PE. PE does not affect hippocampal volume (Rubin et al., 2016). Recent research linked deficits in accurately discriminating context between threating and safe situations to a smaller hippocampus (Negash et al., 2015), thus extinction recall in PE may be affected by it. While a few studies using other exposure-based treatments for PTSD have demonstrated increased hippocampal activation in patients with PTSD following psychotherapy (Felmingham et al., 2007; Peres et al., 2007), no study to date has demonstrated increased activation with PE (Lindauer et al., 2005; van Rooij et al., 2015; Rubin et al., 2016). Overall, the fear and contextualization neurocircuitries (amygdala, vmPFC, ACC and the hippocampus) appear to be heavily involved in the processes of extinction learning and recall in PE. As expected, PE restores the balance in the vmPFC-amygdala loop and decreases the activation in the ACC during extinction recall. Interestingly, the functioning and volume of some of these structures may also be a prognostic indicator for PE’s efficiency and a target for enhancement interventions.

Emotion regulation neurocircuitry is not a unified neurocircuitry but a set of circuits that share regions with the fear neurocircuitry (Ochsner et al., 2002; Sheynin and Liberzon, 2017) but there is some evidence to suggest that activation in and connectivity between these circuits may underlie treatment outcomes in PE. In one neuroimaging study, patients underwent an implicit (unintentional) emotion regulation task and an explicit (intentional and deliberate) emotion regulation task before receiving a course of PE (Fonzo et al., 2017). Individuals with greater vmPFC activation during the implicit emotion regulation task showed larger PTSD symptom reduction at the end of treatment (Fonzo et al., 2017). This points to the possibility that certain individuals’ brains may have diminished capacity to reduce interference from an emotionally-salient cue in the environment, possibly making it more difficult to fully engage in PE. Interestingly, activation during the explicit emotion regulation task at baseline did not predict symptom change (Fonzo et al., 2017). This is consistent with EPT’s emphasis on emotional engagement during exposures to facilitate extinction and inhibitory learning insofar that efforts at attenuating emotional responses during exposure are counterindicated and interfere with learning (Craske et al., 2008; Foa, 2011). Increased competence to cope with negative affect is a type of inhibitory learning speculated to be an active ingredient in PE’s effectiveness (Rauch et al., 2001; Foa and Rauch, 2004; Zalta et al., 2014). These emotion regulation skills are acquired both through successful exposures and through processing that occurs following exposures. In a study examining the effect of PE on emotion regulation skills in a sample of individuals with PTSD (Jerud et al., 2016), PE was associated with clinically meaningful improvements in emotion regulation skills, but a course of sertraline had a similar effect (Jerud et al., 2016). Therefore, it is difficult to determine whether the PE effects on emotion regulation are specific to PE or more generalized to any effective PTSD intervention. One neuroimaging study examined connectivity in the “default mode network” (mPFC, parietal cortex) which has been implicated in attentional control (Fox et al., 2015) before and following a mindfulness based exposure therapy (King et al., 2016). Following exposure therapy (but not present-centered therapy), increased connectivity of the default mode network to the dlPFC was observed, and this increased connectivity was associated with decreased avoidance and hyperarousal symptoms of PTSD (King et al., 2016). This pattern of neural activation suggests increased attentional control, one of the components of emotion regulation (Thompson, 2008; Wilcox et al., 2016), following mindfulness based exposure therapy. Correlation with decreased avoidance and hyperarousal also points to greater deployment of emotion regulation skills at the behavioral level. However, no study has examined the effects of PE on the emotion regulation neurocircuitry, therefore, it is impossible to know how much of the effect is due to mindfulness training and how much is to the exposure component. As suggested previously, emotion regulation deficits may be a transdiagnostic factor contributing to the development and maintenance of various types of psychopathology (Bradley et al., 2011; Sheynin and Liberzon, 2017), therefore, increased connectivity in that region may reflect an alleviation in symptoms due to an effective treatment in general, rather than due to a mechanism specific to PE. PTSD results in a disrupted network of interconnected brain regions. The neuroimaging studies to date suggest that changes in some neurocircuitries are more unique to the putative mechanisms of PE (e.g., the fear and contextualization neurocircuitries are affected by the extinction learning and recall component of PE) while changes in others may be more generally related to mechanisms not unique to PE (e.g., inhibitory learning related to increased emotion regulation skills). Successful extinction recall in PE appears to be related to increased functional coherence between vmPFC, amygdala, and the hippocampus (Helpman et al., 2016a). Similarly, increased connectivity between areas implicated in attentional control (default mode network) and areas implicated in explicit emotion regulation (dlPFC) appears to be indicative of more effective coping with negative affect and downregulation of emotional responses in stressful situations (King et al., 2016; Fonzo et al., 2017). Therefore, while the functioning of individual brain structures is important and clearly impacted by the active components of PE, it appears that increased and more efficient communication between various structures that regulate each other, is of greater importance in PTSD remission.

## Neurobiological Enhancements of PE Treatment

The purpose of this focused review is to create a bridge between neuroscience and practice of PE therapy by examining the effects of exposure therapy on the neural circuits implicated in PTSD. Further, we aim to use the advances in neuroscientific treatment outcome research in order to propose potential enhancement to the practice of PE based on the neurocircuits that have been shown to be affected by it. The neurobiological findings to date may be applied in two ways: (1) to identify potential PE treatment enhancements in order to facilitate emotional engagement, extinction and emotion regulation/inhibitory learning; and (2) to identify individuals who may be more likely to respond to certain enhancement, in order to provide personalized treatment.

Selective serotonin reuptake inhibitors (SSRIs) are recommended in treatment guidelines as treatment for PTSD, following the evidence-based psychotherapies such as PE (Institute of Medicine, 2014; American Psychological Association, 2017). Preliminary evidence suggests that facilitation of serotonergic transmission produced by SSRIs results in increased activation in the vmPFC regions (Brady et al., 2000; Davidson et al., 2001). A recent study compared the effects of PE alone or sertraline alone on attentional inhibition (as measured by a laboratory task) in individuals with PTSD to examine the effects of each of these therapies on one of the purported main mechanism of change in treatment of PTSD, inhibitory learning (Echiverri-Cohen et al., 2016). The authors found that those who showed more symptom improvement with PE treatment showed greater improvements in inhibitory processes from pre- to post-treatment. In contrast, those who showed greater symptom reductions on sertraline made less improvement in their inhibitory processes (Echiverri-Cohen et al., 2016). This discrepancy may point to different mechanism of action of each of these treatment and support the hypothesis that SSRIs bring about more bottom-up neurochemical changes in the fear circuitry, vs. the top-down changes produced through extinction and inhibitory learning in PE. In addition, another study found emotion dysregulation was improved equally as a result of PE or sertraline in individuals with PTSD from pre- to post-treatment but the mechanisms of action of each treatment were not tested (Jerud et al., 2016). Given that SSRIs may have a suspected different path of action than PE on restoring the balance in the limbic-prefrontical system, many have speculated that combining these interventions may augment each alone or alternatively that different people may respond to each treatment. In two studies using reverse designs (one augmenting PE on SSRI non-responders and the other augmenting SSRI for PE non-responders), results supported that for partial responders to SSRI augmentation with PE was effective (Rothbaum et al., 2006). However, when SSRI was added for those who only partially or did not respond to PE, there was no added benefit (Simon et al., 2008). Identification of genetic variants associated with more robust response to SSRIs or PE in PTSD could allow for more personalized and effective treatments. Ongoing biomarkers studies of treatment response (e.g., project PROGrESS; Rauch et al., 2018) promise to inform our field in these areas over the next several years.

Glutaminergic and GABAnergic neurotransmission has been implicated in fear conditioning and extinction (Riaza Bermudo-Soriano et al., 2012), and D-cycloserine (DCS), a partial agonist at the *N*-methyl-D-aspartate (NMDA), has been implicated in fear extinction through the modulation of NMDA receptors in the amygdala (Norberg et al., 2008). While preclinical studies showed promising results (Walker et al., 2002; Ledgerwood et al., 2005; Yang and Lu, 2005) when DCS was used to facilitate extinction learning in rodents, human studies using exposure therapy yielded mixed results. Of studies that examined DCS as an augmentation agent in exposure therapy, only one found improved treatment outcomes with DCS (Difede et al., 2014). Two studies found no noticeable added benefit from supplementing exposure therapy with DCS (de Kleine et al., 2012; Rothbaum et al., 2014), while one study found poorer treatment outcomes in the group who received DCS-augmented exposure therapy compared to placebo (Litz et al., 2012). These mixed findings suggest that DCS may be an effective exposure enhancer for a certain subgroup of individuals with PTSD. For example, de Kleine et al. (2012) found that patients with high initial scores and who completed all treatment sessions actually benefited from DCS augmentation. DCS has also been shown to facilitate reconsolidation (or updating) of fear memory in animal studies (Lee et al., 2006). Therefore, it is possible that in the case of an exposure in which extinction is insufficient, fear memory is reconsolidated in a more intense form (Litz et al., 2012), basically making an unsuccessful exposure worse. To date, the majority of studies using DCS and exposure therapy for PTSD have failed to find a clear enhancing value of DCS. Studies with individuals with fear of heights as well as social anxiety found that administering DCS following a successful exposure, did indeed augment those exposures (Smits et al., 2013a,b; Tart et al., 2013). It appears that DCS may be a very specific intervention and has to be carefully tailored to individual’s symptom severity and their response to IE, and more studies of moderators and mediators of DCS impact on extinction and learning are needed before recommending it as an augment to PE.

Exogenous administration of the neurosteroids DHEA(S) and pregnenolone that modulate GABA action in the brain has also been studied in PTSD interventions. Dehydroepiandrosterone (DHEA) and its sulfated metabolite (DHEAS) are endogenous neurosteroids with negative modulatory effects (GABA antagonist) on the GABAnergic system (Maninger et al., 2009). DHEA(S) levels have been shown to be inversely related to depression (Barrett-Connor et al., 1999; Wong et al., 2011) and positively related to executive function (Alhaj et al., 2006; Davis et al., 2008). Allopregnanolone (ALLO), an endogenous neurosteroid, is one of the most potent GABA agonists (Lambert et al., 2003) and has been shown to have anxiolytic effects (Paul and Purdy, 1992). In a sample of healthy men, single-dose DHEA administration was associated with decreased activation in the amygdala, and increased connectivity between the amygdala and the hippocampus during emotion regulation laboratory task (Sripada et al., 2013b). Administration of pregnanolone was associated with decreased amygdala activation and with increased connectivity between prefrontal cortical regions and the amygdala during that same task (Sripada et al., 2013c). Therefore, it appears that DHEA(S) and ALLO may be involved in the emotion regulation neurocircuitry and affect communication between the amygdala and the prefrontal regions related to executive functioning. Of note, ALLO has been shown to have positive effects on pain tolerance (Scioli-Salter et al., 2016), symptoms of traumatic brain injury (Marx et al., 2016), and depression and bipolar disorder (Osuji et al., 2010; Brown et al., 2014). The same way emotion dysregulation may be a transdiagnostic indicator of emotional disorders, DHEA(S) and ALLO may have a transdiagnostic therapeutic effect, independent of the mechanisms of action of PE and therefore not specific to PTSD (Rasmusson et al., 2017).

As increased neural connectivity within and between different neurocircuits is emerging as an important mechanism of action in psycho- and pharmacotherapies, repetitive transcranial magnetic stimulation (rTMS) has been of interested as a stand-alone and add-on treatment for PTSD (Karsen et al., 2014; Yan et al., 2017). rTMS uses an electromagnetic field to non-invasively stimulate cortical neurons through repeated changes in the coil’s magnetic field (George et al., 2002; George and Post, 2011) and has been approved by the Food and Drug Administration for the treatment of drug-resistant depression. The most common target for these studies has been broadly the dlPFC (Karsen et al., 2014), with its projections to the fear and contextualization circuits (i.e., the amygdala, the hippocampus, and the vmPFC). To date, several reviews and/or meta-analyses have demonstrated the effectiveness of rTMS for treatment of PTSD by targeting the right dlPFC regions (Karsen et al., 2014; Clark et al., 2015; Yan et al., 2017). Fonzo et al. (2017) found that when the right dlPFC was stimulated via rTMS, it downregulated the inhibition of the left amygdala; the magnitude of that effect was a predictor of PE response. A study that used TMS as a stand-alone treatment (no exposure therapy) found that the pre-existing connectivity between the ACC and the default mode network responsible for attentional control as well as connectivity between the amygdala and the vmPFC predicted patient’s response in rTMS treatment (Philip et al., 2018). Therefore, assessing patient’s brain activation patterns pre-treatment may be used as a predictor of treatment response. More importantly, the rTMS studies to date identify neurostimulation-accessible brain regions that may serve as targets for enhancing exposure therapy either prior to or during the course of PE (Fonzo et al., 2017).

One novel candidate enhancement to PE that is purported to target the actual engagement in treatment and the quality of therapeutic alliance is a neuropeptide oxytocin (Olff et al., 2010). Oxytocin’s properties of enhancing prosocial behavior, trust and warmth may be useful in facilitating extinction and inhibitory learning in exposure therapy through successful therapeutic alliance (McLaughlin et al., 2014) and decreasing dropout rates (Tuerk, 2014). One unpublished small study found that a single administration of oxytocin to individuals with PTSD decreased anxiety scores, irritability, intensity of intrusive symptoms, and increased the desire for social contact (Yatzkar and Klein, 2010). Thus far, only one pilot randomized controlled trial examined the efficacy of administering intranasal oxytocin 45 min prior to each IE in patients with PTSD who were undergoing PE (Flanagan et al., 2018). The group who received oxytocin demonstrated lower PTSD and depression symptoms during PE and had higher therapeutic alliance scores but these differences were not statistically significant, potentially due to low power (Flanagan et al., 2018). Therefore, larger RCTs are warranted to further explore the efficacy of oxytocin as a supplement to PE. Another promising novel augmentation to PE is MDMA (Thal and Lommen, 2018), a substituted amphetamine (i.e., a class of compounds based on the amphetamine structure derived by replacing one or more hydrogen atoms in the amphetamine core structure) with properties similar to mescaline, psilocybin, and other psychedelic compounds. The cognitive effects of MDMA in clinical studies have included enhanced mood, happiness, physical and mental relaxation, increased emotional responsiveness, increased openness and extraversion, and increased prosocial behaviors such as trust and feelings of closeness to other people (Harris et al., 2002; Vollenweider et al., 2002). In addition, MDMA has been demonstrated to facilitate extinction retention in mice (Young et al., 2017). The exact pharmacological mechanisms of MDMA’s action are not well-understood. MDMA is known to acutely facilitate the release of serotonin and oxytocin (Dumont et al., 2009; van Wel et al., 2012), potentially contributing to decreased avoidance and greater engagement in exposure-based therapy.

To date, there have been three clinical trials examining the effectiveness of MDMA as an adjunct to psychotherapy for treating PTSD (Mithoefer et al., 2011, 2018; Oehen et al., 2013). In all three of these studies, MDMA was administered shortly before the psychotherapy session and participants demonstrated significant decreases in PTSD symptoms at the end of treatment and at follow up (Mithoefer et al., 2011, 2018; Oehen et al., 2013). Of note, psychotherapy administered in these studies was not one of the empirically supported exposure-based treatments for PTSD and was instead non-directive and focused more on experiencing than on verbal exchanges (Mithoefer, 2011). MDMA remains to be tested as an enhancement to PE. Given the proposed mechanism of action in MDMA (i.e., increases in serotonin and oxytocin, increased trust and openness to new ideas), it is possible that it would be particularly helpful to apply it during the processing portion of the session, when patients’ beliefs about themselves, others, and the world are reflected and become less rigid. Alternatively, MDMA may be useful only for those patients who exhibit slow or no extinction during IE. One recent study found that, on a personality trait level, patients who received MDMA during psychotherapy exhibited an increase in the Openness trait post-treatment and at follow-up, while their Neuroticism trait remained unchanged (Wagner et al., 2017). This suggests that indeed the mechanism of effective action in the case of MDMA may be greater openness to new ideas and decreased cognitive rigidity rather than decreased negative emotionality. While promising, the efficacy and effectiveness of supplementing PE with MDMA remains an empirical question.

## Future Directions and Conclusions

The mechanisms of action of PE have been of great interest in the past decade and neuroimaging studies followed suit. Currently, it is clear that various neural circuits are impacted in the course of PE but it also appears that certain patterns of neural activation and connectivity predict patient’s response in PE. Generally, exposure therapy appears to have an impact on the fear and contextualization neurocircuitry by facilitating improved communication between the vmPFC, hippocampus, and the amygdala, leading to downregulation of the fear response in the amygdala (Helpman et al., 2016a). PE has been shown to decrease the activity in and the volume of the ACC. This increased coherence between these particular regions may be the mechanisms unique to PE (or exposure therapy in general) given that successful exposures lead to extinction learning and recall, thus extinguishing the overgeneralized fear response. There appears to be a more general process of emotional regulation that is of importance as well. Similarly, increased connectivity between areas implicated in attentional control (default mode network) and areas implicated in explicit emotion regulation (dlPFC) appears to be indicative of more effective coping with negative affect and downregulation of emotional responses in stressful situations (King et al., 2016; Fonzo et al., 2017). This process has been conceptualized as more transdiagnostic and less specific to PTSD or PE. Finally, it appears that neural connectivity *prior* to treatment may have profound impact on treatment response, offering some directions for future use of prognostic indicators as well as enhancements.

One future direction concerns identifying the types of patients who would most benefit from PE (and those who might not). Larger hippocampal volume has been shown to be associated with better outcomes in PE (Rubin et al., 2016). Increased connectivity between the fear and the emotion regulation neurocircuitries during emotionally salient tasks is also a predictor of treatment success is PE (Fonzo et al., 2017). Therefore, neuroimaging or electroencephalography should be used in future studies to not only corroborate these findings but also potentially establish certain cut-off benchmark for the magnitude of connectivity or the relative size of the hippocampus in optimal PE response. Given consistently positive findings regarding the effectiveness of rTMS in PTSD symptom improvement, it is a priority to continue to research this potential PE enhancement. Specifically, future studies should focus on neurobiological and psychological moderators and mediators of rTMS effects on PE response, as well as on identifying those individuals who would most benefit from rTMS. Currently, it appears that rTMS may be of particular importance for those people whose brains do not “let” them engage fully in exposure therapy, i.e., those with pre-existing decreased connectivity between the key neurocircuitries, however, that is an empirical question.

Further examination of the enhancements that promote the sense of connectedness and self-compassion (i.e., oxytocin and MDMA) is also warranted. Currently, it is unclear whether these agents affect neural circuits that are more unique to exposure therapy (such as the fear or the contextualization circuits) or the transdiagnostic emotion regulation circuitry, or another circuitry altogether. They may be promoting decreased behavioral avoidance (Dumont et al., 2009; van Wel et al., 2012) but neuroimaging studies examining these agents’ effects on neurocircuitries that are of most importance in PE would clarify that conjecture. Concurrently, studies examining the effects of these agents on PE response would be helpful in clarifying whether these agents offer any added benefit above and beyond regular PE. If so, then identifying patients who would be appropriate candidates for such enhancements would be the logical next steps, especially given the heterogeneity of PTSD symptoms.

In order to tap into the change in the ability to handle and regulate negative emotions and identify individuals most likely to benefit from treatment, future studies should employ various emotion regulation tasks during neuroimaging scans pre- and post-treatment. For instance in the PROGrESS trial (Rauch et al., 2018), participants engage in an emotional faces matching task aimed at isolating the amygdala reactivity to threat (i.e., angry and fearful faces) and non-threat (i.e., happy and neutral faces; Hariri et al., 2002). Additionally, participants engage in the Emotion Regulation Task designed to measure both, the explicit emotion regulation (Gyurak et al., 2011) activation in the prefrontal cortical regions including dlPFC and vmPFC, as well as the implicit emotion regulation manifested by the activation in vmPFC and the amygdala (Costafreda et al., 2008; Buhle et al., 2014). Finally, the implicit emotional regulation processes (Gyurak et al., 2011) are also measured using attentional control with emotional faces task (SEAT; Sripada et al., 2013a). Administration of such tasks while undergoing neuroimaging pre- and post-PE is essential in identifying not only connectivity changes resulting from treatment in the fear, contextualization, and emotion regulation neurocircuitries but also pre-existing connectivity patterns that may be indicative of individuals who might require enhancements in order to fully benefit from PE. It would be useful to develop and utilize neuropsychological measures of hippocampus activity in order to evaluate PE’s effect on its function. Additionally, salivary and plasma concentrations of DHEA(S) and ALLO have been shown to be associated with increased communication between prefrontal cortical regions and amygdala (Sripada et al., 2013c). Therefore, establishing benchmarks for the extent of connectivity related to certain concentrations of DHEA(S) and ALLO, and measuring these concentrations pre and during PE may be a potentially less burdensome method of identifying increased communication between the relevant brain structures.

While most of our focus has been on improving response to PE, retention, as previously mentioned, is a key area for improvement across PTSD interventions (Tuerk, 2014). Neurobiological advances can also provide insights that drive better retention through more personalized, more efficient, and more effective care. Dropout rates across populations and treatment setting are approximately 30% or more (Bradley et al., 2005; Eftekhari et al., 2013), which is not surprising given that PTSD is characterized by behavioral avoidance. Therefore, further exploring the effectiveness and the mechanisms of action of oxytocin and MDMA as enhancements to PE is warranted in an effort to improve retention in PE. For instance, for those who have higher potential for dropout, administration of intranasal oxytocin may be particularly effective in retaining them in treatment through increased connectedness with their PE provider. Identifying neurobiological biomarkers of those who are at risk of dropping out would aid in clinical decision-making process regarding patients who are good candidates for such an enhancement.

## Conclusions

In this focused review, we reviewed the neurobiological mechanisms underlying the effectiveness of PE, an empirically-supported efficacious treatment for PTSD, and the enhancements that can be applied to increase treatment response and retention. One of the proposed mechanisms of action in PE is exposure which facilitates extinction of a fear response and new adaptive learning. Neurobiologically, PE and successful extinction recall has been associated with increased neural connectivity between vmPFC, amygdala and the hippocampus. Increased connectivity in regions implicated in emotion regulation has also been shown to result from PE although it appears that this change in activation is more transdiagnostic and not unique to PE. Since neural connectivity and communication seem to be at the heart of symptom alleviation in PTSD, treatment enhancements that promote such connectivity, particularly rTMS, offer the most appropriate targets for future research into effectiveness of PE. Further research into the effects of oxytocin and MDMA on treatment response and retention is also warranted. Finally, establishing neurobiological benchmarks for identifying individuals who are less likely to benefit from treatment would be an exciting development to help guide clinical decision-making as to who should receive enhancements to PE.

## Author Contributions

MS involved in planning, lead author, primary writer and editor. LM involved in planning, led the literature review, wrote sections and edited. SR senior author involved in planning, review, writing and editing.

## Conflict of Interest Statement

The authors declare that the research was conducted in the absence of any commercial or financial relationships that could be construed as a potential conflict of interest. The reviewer JA and handling Editor declared their shared affiliation at the time of the review.

## Footnotes

^1^We have chosen to use extinction rather than the term habituation used by Foa and Kozak (1986) based on more recent theorists and neuroscience research that aligns more with extinction processes (see Rauch and Liberzon, 2016 for review).
